# Quality of Life of Patients after Kinesio Tape Applications Following Impacted Mandibular Third Molar Surgeries

**DOI:** 10.3390/jcm10102197

**Published:** 2021-05-19

**Authors:** Aleksandra Jaroń, Olga Preuss, Berenika Konkol, Grzegorz Trybek

**Affiliations:** Department of Oral Surgery, Pomeranian Medical University in Szczecin, Powstańców Wielkopolskich 72/18, 70-111 Szczecin, Poland or aleksandra.jaron@pum.edu.pl (A.J.); olga.preuss@pum.edu.pl (O.P.); berenika68@gmail.com (B.K.)

**Keywords:** pain, wisdom tooth removal, complications, kinesiotaping, oral surgery, quality of life

## Abstract

Today, extraction of the impacted third molar is the most common procedure performed in oral surgery departments. One of the methods currently investigated—in terms reducing the severity of non-infectious complications and decreasing Quality of Life following third molar surgeries—is Kinesio Taping (KT). The aim of the study was to evaluate the impact of Kinesio Tape application on Quality of Life. A total of 100 asymptomatic patients with impacted third lower molar were included. The study participants were randomly divided into two groups: a study group with the application of KT (n = 50) and a control group (without KT) (n = 50). Removal of the impacted third lower molar was performed in each patient in a standardized fashion. For assessment of Quality of Life, the modified University of Washington Quality of Life Questionnaire (UW-QoL v4) was used. Patients with Kinesio Tape application scored higher in all domains. Statistically significant differences between the two groups were found in the following domains: “Activity”, “Mood”, “Health-related QoL during the past 7 days” and “Overall QoL during the past 7 days”. There were no significant differences in significant problems and important issues between groups. Kinesio Taping has a significant impact on Quality of Life after impacted third molar removal. It should be considered as one of the noninvasive methods to reduce postoperative non-infectious complications.

## 1. Introduction

Extraction of the impacted third molar (ITM) is the most common procedures performed in oral surgery departments [1,2,3,4]. After the procedure, the sequelae of non-infectious postoperative complications often occur, and these include trismus, post-extraction pain and edema. Their occurrence is more frequent following extraction of the lower ITM [1,2,3]. Even though typically not health-endangering, their frequency constitutes a major therapeutic problem which may significantly reduce the patients’ quality of life (QoL) after surgery [5].

QoL is defined as a patient’s perception of the impact of their disease or treatment, or both, on their daily life and their physical, psychological, and emotional wellbeing [6]. The basic methods of QOL assessment are various questionnaires which occasionally include pain scales, e.g., VAS—Visual Analogue Scale [7,8,9,10]. There is a number of studies assessing QoL after the removal of ITM. The majority of cases indicate a reduction in QOL during the recovery period.

Currently, several pharmacological and non-pharmacological methods are available to help to avoid or minimize these complications, and they mostly include corticosteroids or NSAIDs (nonsteroidal anti-inflammatory drugs), cryotherapy and LLLT (low-level laser therapy) [11,12,13,14]. Recently, it has been demonstrated that Kinesio Taping (KT) reduces the severity of non-infectious complications following third molar surgery [15,16,17]. According to some studies, this method may decrease post-extraction pain and trismus as well as may reduce the size of facial edema. The KT method was invented by Kenzo Kase in Japan in 2003 [18], and it is based on the application of Kinesio Tapes—thin, waterproof, adhesive, elastic tapes. The use of KT leads to the activation of mechanoreceptors, thus increasing the excitability of muscles by the central nervous system—by causing pressure, stretching the skin, and improving blood and lymph circulation by pulling the subcutaneous tissue and skin from the muscles [19]. KT is a non-invasive, hypoallergenic method which has a 24-h therapeutic effect. To the best of our knowledge, reports on the application of KT after third molar surgery are few and there is no single study investigating their impact on QoL.

The aim of the study was to evaluate the potential impact of KT on QoL in patients after surgical extraction of the impacted lower third molar.

## 2. Materials and Methods

The study was approved by the Bioethical Committee (KB-0012/152/13-KB-0012/135/15). A total of 100 healthy, asymptomatic patients with at least one impacted third lower molar were included in the study. Impaction was evaluated on panoramic x-rays and indications for extraction were orthodontic reasons or previous pericoronitis. All cases were unilateral. Exclusion criteria included: any general diseases, patients under 18 years of age, pregnancy, lactation and tobacco smoking. Study participants were randomly divided into two parallel groups: a study group with the application of KT (n = 50) and a control group (without KT) (n = 50). One hundred opaque and sequentially numbered envelopes were used for concealment of allocation to trial groups. Each envelope contained a group assignment for one patient, determined in advance by a random number table. Extraction was performed under local anaesthesia using the same, standardized procedure. The block anaesthesia was made with 2 ampoules of 2% lidocaine with noradrenaline 1:80,000. All patients were asymptomatic and did not receive preoperative therapy. The standard protocol included buccal access and bone removal with rotary instruments. After extraction, the wound was rinsed with a sterile solution of physiological saline, and after achieving local hemostasis, the sutures were made. Because the Quality of Life questionnaire is somewhat subjective, patients in both the study and control groups were treated with ketoprofen in a 100 mg dose taken twice daily after surgery to give the assurance that they were under medical care.

**Figure 1:**
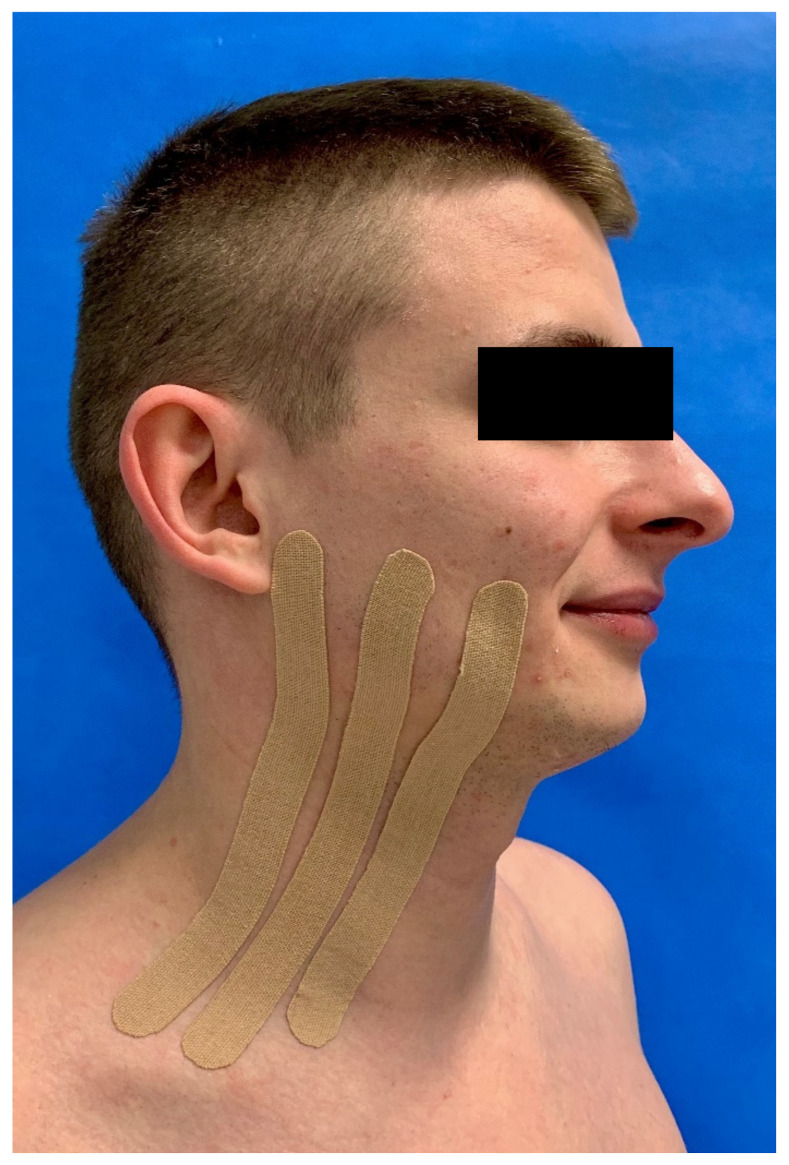
Kinesio Tape application.

K-Active Tape Classic (Nitto Denko Corporation, Japan, distribution: K-Active Europe GmbH, Germany) was applied to patients from the study group immediately after surgery. The length of the tape was individually adapted to each patient. KT was applied using the lymphatic technique to a patient’s face [15], extending from the supraclavicular region to line where the largest swelling was suspected (Figure 1). The 5 cm tape was divided into 3 parts. It was recommended to keep the tapes for the next 5 postoperative days. Sutures were removed 7 days after surgery.

For the assessment of QoL, the modified University of Washington Quality of Life Questionnaire (UW-QoL v4) was used (Table 1, Table 2, Table 3, Table 4 and Table 5). This head and neck cancer Health-Related Quality of Life Questionnaire was adapted to patients after third molar surgery [20]. The modified University of Washington Quality of Life Questionnaire (UW-QoLv4) consisted of 12 domains and 4 questions. This questionnaire focuses on current patient health and QoL within the past 7 days (Table 1, Table 2, Table 3 and Table 4) Its modification consisted of alteration in domains No. 8 and 13, in which the word “shoulder” was replaced with the word “face”, and, in domains No. 11 and 12, the word “cancer” was replaced with the word “operation”. These modifications resulted in better adaptation to oral surgery patients. Every patient was asked to fill in UW-QoL v4 before the procedure and in the 7th postoperative day.

Power was calculated using the method described by Shieh et al. and the R package “wmwpow” [21]. (Shieh, G., Jan, S.L., Randles, R.H. (2006). On power and sample size determinations for the Wilcoxon–Mann–Whitney test. Journal of Nonparametric Statistics, 18(1), 33–43.) Shieh power was 80% assuming 5% alpha and an effect size (given as the probability that outcomes were different between the groups) of 66% (two-sided).

Statistical analysis was carried out using the statistical R package. The normality of the distribution of variables was assessed using the Shapiro–Wilk test. The Mann–Whitney test was implemented for comparative analysis of quantitative variables of the study groups in the absence of normality of their distribution. The qualitative variables between both groups were compared using the chi-square test. For the analysis of qualitative variables in the 2 × 2 tables, a chi-square test with Yates correction was employed. In the case where low values were expected in the tables (<5), Fisher’s exact test was used. In all calculations, a *p*-value of less than 0.05 was considered significant.

## 3. Results

### 3.1. Group Characteristics

The number of patients who took part in the study totalled 100. The study group consisted of 36 females and 14 males, while the control group consisted of 38 females and 12 males. The age of patients in the study group ranged from 19 to 59 years, while, in the control group, the range was between 18 and 38 years. The time of the procedure was similar in both groups (*p* = 0.801). The groups did not differ significantly in terms of age and sex (*p* > 0.05). Likewise, the time of the procedure measured in minutes did not show significant differences (Table 5). None of the patients in the study reported side effects after using KT.

### 3.2. QOL Results

**Figure 2:**
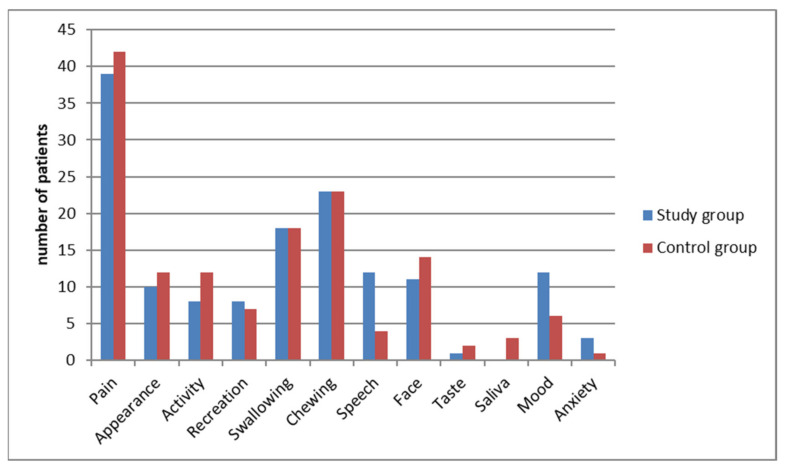
Important issues for patients during recovery after removal of ITM.

**Figure 3:**
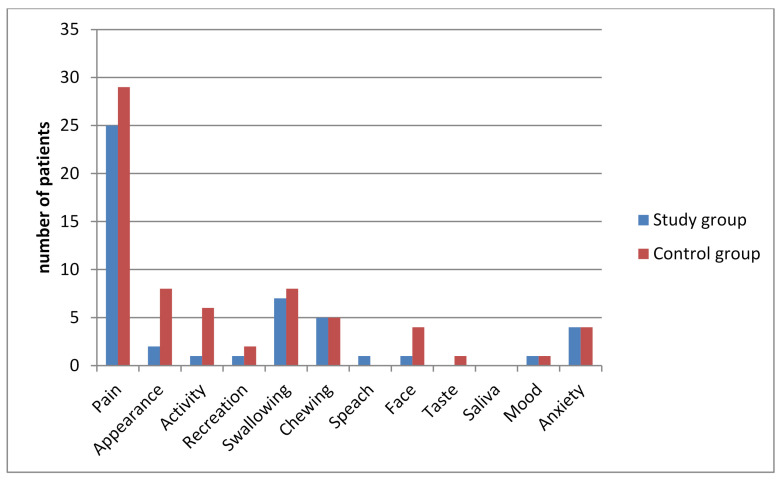
Significant problems for patients during recovery after removal of ITM.

We discovered significant differences between the two groups in the following domains: “Activity”, “Mood”, “Health-related QOL during the past 7 days” and “Overall QOL during the past 7 days”. Patients with KT application scored higher in the above domains (Table 6). There was also a significant decrease in overall QOL during the seven postoperative days in patients of the control group (Table 7). Interestingly, we did not reveal significant differences between the study groups in terms of choosing important issues and significant problems. Patients of both groups indicated that the most important issue and problem during the seven postoperative days were pain sensations. The last question in the questionnaire concerned issues that were most important to the patient during the last 7 days (Figure 2). Every patient was asked to indicate up to three issues. Significant problems were evaluated using specific algorithms to calculate the score. They use information from domain scores and the question of the important issues (Table 6; Figure 3).

## 4. Discussion

The concept of postoperative quality of life determines the impact of surgical procedures on the change in everyday functioning activity and the degree of postoperative pain. Previous studies have demonstrated a decrease in the QoL after third molar surgery as well as after routine teeth extractions [22,23]. However, to the best of our knowledge, there were no studies which implemented University of Washington Quality of Life Questionnaire (UW-QoLv4) for QoL assessment. The typically employed questionnaires include the 14-item Oral Health Impact Profile (OHIP-14), the 16-item UK Oral Health-related Quality of Life measure (OHIP-16) (The Oral Health Impact Profile-16), OHQOLUK-16 (United Kingdom Oral Health-related Quality of Life measure questionnaire -16), EQ 5D-3L (The EuroQol Group questionnaire—5 Dimensions and 3 Levels), OHRQoLUK (United Kingdom Oral Health-related Quality of Life instrument), and PoSSe (Postoperative Symptom Severity questionnaire) [7,10,22,23,24,25,26,27,28]. EQ 5D-3L studies showed improvement in postoperative QoL after seven days but with a significant decrease immediately after the surgery [6]. This questionnaire does not inquire about health before operation; thus, no comparison with the preoperative period is possible. By contrast, in our study, the QoL questionnaire was employed also before treatment, and that allowed for comparison of health status before and after the surgery.

Moreover, the questionnaire we use is reliable, valid, sensitive and precise [18]. Validity of the questionnaire is confirmed by the substantial number of publications that use UW-QoL v4. We decided to use UW-QoLv4 because it is validated, simple to process, and proven to provide clinically relevant information about the patient’s physical and mental condition [29,30].

According to Deepti, QoL decreases within a 5-day postoperative period [7]. However, between the 6th and 7th postoperative days, the decrease was found to not be statistically important. In the present study, application of Kinesio Tape lasted 5 days and it was assumed that 7 days are required to return to the proper daily activity.

According to the results of the present study, two domains were not important problems in both groups since the most important issues and problems were: “pain”, “appearance” and “activity”. In the study by Raymond involving 630 patients, the surgery affected the QoL of approximately half of the patients and the most important issue for them was compromised oral function and pain. In the current series, apart from “pain”, “swallowing” and “chewing” were also the most important issues for patients of both groups. Likewise, McGrath noticed a significant decrease in QoL after third molar surgery in the immediate postoperative period [31]. In turn, Grossi revealed that the most affected domains were “eating” and “interference with daily activities” [32]. In addition, in Braimah’s study, the significantly affected domains were “eating”, “laughing”, “smiling”, “work”, and “speech” [33]. The difference between the results of studies for specific domains may be the cause of various populations appraised. The geographical aspect should be taken into consideration and its relationship to important issues chosen by patients.

In the studies of Ristow et al. [15,34,35,36] on the impact of surgical removal of the impacted mandibular third molar, surgical treatment of the zygomatic-orbital and mandibular fractures on patients’ quality of life, the assessment was made by asking four questions to the patients of the study group and two questions to the control group. The questions concerned the patient’s satisfaction after the procedure and the evaluation of facial swelling. In the group with the applied KT, the patients were asked if the KT disturbed their daily functioning and if their presence inhibited the possibility of head movement. It was, therefore, less extensive than the questionnaire used in the self-examination, which consisted of more questions and was used in the same form both in the examined and control group.

Yurttutan et al. [37] compared different treatments for patients with mandibular third molars and mild pericoronitis. Treatment by extraction or by a periodontal approach and its effect on QoL were compared. Tooth extraction, long term, was more effective than periodontal treatment. Unfortunately, the effect of KT tide on QoL was not assessed.

Ibikunle demonstrated that injection of prednisolone or cryotherapy after third molar surgery resulted in a lesser decrease in QoL in comparison with the control group. The domains “ability to chew”, “ability to swallow” and “diet change” were most often reported as affected and the scores were significantly higher in patients without prednisolone injection [27,29]. Likewise, dexamethasone injections used in the study by Deo caused lower deterioration of QoL as compared to the control group. Although changes in the domains “speech”, “eating”, “sickness” and “interference with daily activity” were recorded, significant differences were demonstrated only in domain “sickness” [38].

Similarly, LLLT has an impact on pain intensity and swelling problems after third molar surgery. In a study by Bantinjan, where no questionnaire was used, patients subjected to the laser therapy indicated their symptoms as less nagging then in the control group [39]. The study by Colorado-Bonnin et al. showed that over 50% of participants had to withdraw from their work for the period of convalescence, and as the main reason for the decrease in their QOL, they indicated pain and edema, which led to their social isolation. Over 40% of patients did not maintain their standard activity. Patients often also refrained from practicing sports and other hobbies (68.1%) [8]. Likewise, in the present study “activity” and “mood” on the 7th day were significantly higher in patients with KT than in the control group as well as the difference between questionnaire filled on the seventh day and pre-operative period in “Overall QoL during the past 7 days” and “activity”.

Although the results of the study are promising, KT improves postoperative QoL compared to the no KT application, the study has some limitations. Although the study group was not very numerous (100 people), according to the authors’ knowledge it was the largest of the reported studies so far. In addition, the study could be extended by determining the degree of retention of the mandibular third molar, as well as classification of the position of the impacted tooth in relation to IAN. It is difficult to obtain a blinded control and study group due to the fact that KT tapes are applied to the patient’s skin and are visible.

The QoL assessment questionnaire included a question of appearance. However, it was only determined on the basis of the patient’s subjective evaluation. Volumetric measurements using extraoral 3D scanners and a comparison of color maps of the obtained triangle mesh in the form of an stl file can minimize the risk of measurement inaccuracies and errors. Therefore, the next step in our research will be the measurement of this variable by means of a three-dimensional analysis of the obtained triangle mesh in the face scanning process and reference of the results to postoperative QoL [40,41,42].

In addition, the study of the quality of life of patients after surgical removal of a detained third molar in the mandible is a subjective study. The results represent only the subjective feelings of the patient [43]. QoL questionnaires are a tool to standardize results.

In our study, the removal of impacted mandibular third molar was performed unilaterally. The examination of two types of therapies in one patient makes it possible to compare their therapeutic effect. Each patient feels the intensity of the pain differently, so a split-mouth study would provide the best results [44]. However, it should be kept in mind that in split-mouth tests, the removal of teeth on the right and left side cannot be conducted at the same time, as this will affect the patient’s subjective feelings.

## 5. Conclusions

Proper postoperative care, reduction in postoperative pain and monitoring of the decrease in quality of life have become one of the main goals of modern dental surgery. Therefore, it is important to seek for non-invasive methods to reduce the level of postoperative non-infectious complications. The use of KT has a significant impact on improving the quality of life of patients after surgical extraction of the lower wisdom tooth in the following areas: “Overall quality of life”, “Quality of health in the last seven days”, “Mood” and “Activity”. The use of KT significantly affects the quality of life of patients, between the day of surgical extraction of the lower wisdom tooth and the seventh postoperative day, in the following areas: “Overall quality of life” and “Activity”. Patients with Kinesio Tape had better QoL than in the control group and its application did not disturb their daily activities. The presented results suggest that Kinesio Taping allows improvement in QoL after third molar removal.

## Figures and Tables

**Table 1:** Questionaire’s domains.

Pain □ I have no pain. (100) □ There is mild pain not needing medication. (75) □ I have moderate pain—requires regular medication. (50) □ I have severe pain controlled only by prescription medicine. (25) □ I have severe pain, not controlled by medication. (0) Appearance □ There is no change in my appearance. (100) □ The change in my appearance is minor. (75) □ My appearance bothers me but I remain active. (50) □ I feel significantly disfigured and limit my activities due to my appearance. (25) □ I cannot be with people due to my appearance. (0) Activity □ I am as active as I have ever been. (100) □ There are times when I can’t keep up my old pace, but not often. (75) □ I am often tired and have slowed down my activities although I still get out. (50) □ I don’t go out because I don’t have the strength. (25) □ I am usually in bed or chair and don’t leave home. (0) Recreation □ There are no limitations to recreation at home or away from home. (100) □ There are a few things I can’t do but I still get out and enjoy life. (75) □ There are many times when I wish I could get out more, but I’m not up to it. (50) □ There are severe limitations to what I can do, mostly I stay at home and watch TV. (25) □ I can’t do anything enjoyable. (0) Swallowing □ I can swallow as well as ever. (100) □ I cannot swallow certain solid foods. (70) □ I can only swallow liquid food. (30) □ I cannot swallow because it “goes down the wrong way” and chokes me. (0) Chewing. □ I can chew as well as ever. (100) □ I can eat soft solids but cannot chew some foods. (50) □ I cannot even chew soft solids. (0) Speech. □ My speech is the same as always. (100) □ I have difficulty saying some words but I can be understood over the phone. (70) □ Only my family and friends can understand me. (30). □ I cannot be understood. (0) Face □ I have no problem with my face. (100) □ My face is stiff but it has not affected my activity or strength. (70) □ Pain or weakness of my face has caused me to change my work/hobbies. (30) □ I cannot work or do my hobbies due to problems with my face. (0) Taste □ I can taste food normally. (100) □ I can taste most foods normally. (70) □ I can taste some foods. (30) □ I cannot taste any foods. (0) Saliva □ My saliva is of normal consistency. (100) □ I have less saliva than normal, but it is enough. (70) □ I have too little saliva. (30) □ I have no saliva. (0) Mood □ My mood is excellent and unaffected by the operation. (100) □ My mood is generally good and only occasionally affected by the operation. (75) □ I am neither in a good mood nor depressed about the operation. (50) □ I am somewhat depressed about the operation. (25) □ I am extremely depressed about the operation. (0) Anxiety □ I am not anxious about the operation. (100) □ I am a little anxious about the operation. (70) □ I am anxious about the operation. (30) □ I am very anxious about the operation. (0)

**Table 2:** Important issues.

13. Which issues have been the most important to you during the past 7 days? Tick up to 3 boxes.		
□ Pain	□ Swallowing	□ Taste
□ Appearance	□ Chewing	□ Saliva
□ Activity	□ Speech	□ Mood
□ Recreation	□ Shoulder	□ Anxiety

**Table 3:** Questionnaire’s global questions.

General Questions: Compared to the month before you developed cancer, how would you rate your health-related quality of life? (Tick one box:) □ Much better (100) □ Somewhat better (75) □ About the same (50) □ Somewhat worse (25) □ Much worse (0) In general, would you say your health-related quality of life during the past 7 days has been: (Tick one box:) □ Outstanding (100) □ Very good (80) □ Good (60) □ Fair (40) □ Poor (20) □ Very poor (0) Overall quality of life includes not only physical and mental health, but also many other factors, such as family, friends, spirituality, or personal leisure activities that are important to your enjoyment of life. Considering everything in your life that contributes to your personal well-being, rate your overall quality of life during the past 7 days. □ Outstanding (100) □ Very good (80) □ Good (60) □ Fair (40) □ Poor (20) □ Very poor (0)

**Table 4:** Significant problem—key for scoring [18].

Significant Problem	Scores
Pain, appearance, activity, recreation, mood	0, 25, 50 with important issue (question 13)
Swallowing, speech, anxiety	0, 30
Shoulder, taste, saliva	0, 30 with important issue (question 13)
Chewing	0

**Table 5:** Study groups characteristic.

Baseline Characteristic	Age (Years)		Procedure Time (Minutes)		Sex			
					Female		Male	
	Median	Range	Median	Range	n	%	n	%
Study group (n = 50)	26.5	19–59	21	10–60	36	72	14	28
Control group (n = 50)	25	18–38	24.5	6–60	38	76	12	24
Total (n = 100)	25.5	18–59	23	6–60	74	74	26	26
*p*-value ^1^	0.221		0.801		*p*-value ^2^ 0.82			

^1^ Mann–Whitney test ^2^ Chi-square test.

**Table 6:** The comparative analysis of QOL between the study groups during 7 postoperative days.

Domain	Group	n	Mean	SD	Min	Q1	Median	Q3	Max	*p* *
Pain	study	50	53	27.03	0	25	50	75	100	0.287
	control	50	47.5	26.85	0	25	37.5	75	100	
Appearance	study	50	68.5	25.16	25	50	75	100	100	0.249
	control	50	62	27.77	25	31.25	75	75	100	
Activity	study	50	81.5	21.91	0	75	75	100	100	0.017
	control	50	68	29.47	0	50	75	100	100	
Recreation	study	50	78	23.5	25	75	75	100	100	0.346
	control	50	70	31.94	0	50	75	100	100	
Swallowing	study	50	83	25.01	30	70	100	100	100	0.366
	control	50	79.2	25.54	30	70	100	100	100	
Chewing	study	50	59	29.78	0	50	50	100	100	0.351
	control	50	54	26.42	0	50	50	50	100	
Speech	study	50	89.6	16.28	30	70	100	100	100	0.734
	control	50	89.2	14.55	70	70	100	100	100	
Face	study	50	73.6	27.98	0	70	70	100	100	0.659
	control	50	69.8	31.53	0	40	70	100	100	
Taste	study	50	91.4	15.65	30	77.5	100	100	100	0.154
	control	50	82.2	27.65	0	70	100	100	100	
Saliva	study	50	95	13.59	30	100	100	100	100	0.625
	control	50	94.6	11.64	70	100	100	100	100	
Mood	study	50	74	18.87	25	75	75	75	100	0.048
	control	50	65	23.15	25	50	75	75	100	
Anxiety	study	50	81.2	21.06	30	70	70	100	100	0.863
	control	50	80.6	20.94	30	70	70	100	100	
Physical function	study	50	81.08	15.39	30.83	73.33	82.92	91.67	100	0.24
	control	50	76.87	16.84	42.5	63.75	78.33	87.5	100	
Social-Emotional Function	study	50	73.46	18.24	25	64	79	89	100	0.188
	control	50	67.18	22.63	11	49.25	70	87.75	100	
Health-related QoL compared to time before surgery	study	50	63	27.31	0	50	50	75	100	0.509
	control	50	67	28.32	0	50	62.5	100	100	
Health-related QoL during the past 7 days	study	50	56	18.52	0	40	60	60	80	0.021
	control	50	46.8	20.84	0	40	40	60	80	
Overall QoL during the past 7 days	study	50	64.4	19.5	20	60	60	80	100	0.003
	control	50	52.8	19.7	20	40	60	60	100	

* Mann–Whitney test; *n*—number of patients; SD—standard deviation; Min—minimum value; Max—maximum value; Q1—first quartile; Q3—third quartile; *p*—significance level.

**Table 7:** Changes in QoL between 7th postoperative day and pre-operative period.

Domain	Group	n	Mean	SD	Min	Q1	Median	Q3	Max	*p* *
Pain	study	50	39.5	27.71	0	25	37.5	75	75	0.321
	control	50	45	27.66	0	25	50	75	75	
Appearance	study	50	28	25.58	−25	0	25	50	75	0.176
	control	50	36	28.64	0	0	25	50	75	
Activity	study	50	13	24.35	−50	0	0	25	100	0.045
	control	50	25	29.01	−25	0	25	50	100	
Recreation	study	50	16.5	26.54	−25	0	0	25	75	0.544
	control	50	22	32.2	−25	0	0	50	100	
Swallowing	study	50	16.4	25.05	0	0	0	30	70	0.363
	control	50	20.2	25.67	0	0	0	30	70	
Chewing	study	50	35	30.72	0	0	50	50	100	0.112
	control	50	44	27.92	0	50	50	50	100	
Speech	study	50	8.6	17.84	−30	0	0	30	70	0.611
	control	50	9.6	16.53	−30	0	0	30	30	
Face	study	50	24.6	26.2	0	0	30	30	100	0.8
	control	50	27.8	32.41	−30	0	30	37.5	100	
Taste	study	50	6.2	12.6	0	0	0	0	40	0.143
	control	50	16	28.43	−30	0	0	30	100	
Saliva	study	50	4.4	11.1	0	0	0	0	40	1
	control	50	4.2	12.14	−30	0	0	0	30	
Mood	study	50	10	19.56	−25	0	0	25	50	0.064
	control	50	19.5	26.39	−25	0	25	25	75	
Anxiety	study	50	13.4	22.73	−30	0	0	30	70	0.882
	control	50	13.2	24.94	−70	0	0	30	70	
Physical function	study	50	16.43	14.09	−5	4.17	16.67	23.12	53.33	0.138
	control	50	21.67	16.44	−5	9.37	19.58	35	57.5	
Social-Emotional Function	study	50	21.4	18.05	0	7	15,5	35.5	70	0.332
	control	50	26.6	22.51	−14	7	26.5	43.75	81	
Health-related QoL compared to time before surgery	study	50	−2	30.66	−50	−25	0	0	75	0.137
	control	50	−12	37.88	−100	−50	0	0	100	
Health-related QoL during the past 7 days	study	50	15.6	22.6	−20	0	20	20	100	0.116
	control	50	22.4	27	−40	5	20	40	80	
Overall QoL during the past 7 days	study	50	10.8	22.57	−40	0	10	20	60	0.028
	control	50	20.8	21.37	−20	0	20	40	60	

* Mann–Whitney test; *n*—number of patients; SD—standard deviation; Min—minimum value; Max—maximum value; Q1—first quartile; Q3—third quartile; *p*—significance level.

## Data Availability

The data presented in this study are available on request from the corresponding author.
